# Anionic Hyperbranched Amphiphilic Polyelectrolytes as Nanocarriers for Antimicrobial Proteins and Peptides

**DOI:** 10.3390/ma16247702

**Published:** 2023-12-18

**Authors:** Anastasia Balafouti, Aleksander Forys, Barbara Trzebicka, Angelica Maria Gerardos, Stergios Pispas

**Affiliations:** 1 Theoretical and Physical Chemistry Institute, National Hellenic Research Foundation, 48 Vassileos Constantinou Ave., 11635 Athens, Greece; ampalaf@eie.gr (A.B.); amgerar@eie.gr (A.M.G.); 2 Department of Chemistry, National and Kapodistrian University of Athens (NKUA), 15784 Athens, Greece; 3 Centre of Polymer and Carbon Materials, Polish Academy of Sciences, 34 ul. M. Curie-Skłodowskiej, 41-819 Zabrze, Poland; aforys@cmpw-pan.pl (A.F.); btrzebicka@cmpw-pan.pl (B.T.)

**Keywords:** amphiphilic copolymers, hyperbranched, polyelectrolytes, self-assembly, protein complexation, nanoparticles

## Abstract

This manuscript presents the synthesis of hyperbranched amphiphilic poly (lauryl methacrylate-co-tert-butyl methacrylate-co-methacrylic acid), H-P(LMA-co-tBMA-co-MAA) copolymers via reversible addition fragmentation chain transfer (RAFT) copolymerization of tBMA and LMA, and their post-polymerization modification to anionic amphiphilic polyelectrolytes. The focus is on investigating whether the combination of the hydrophobic characters of LMA and tBMA segments, as well as the polyelectrolyte and hydrophilic properties of MAA segments, both distributed within a unique hyperbranched polymer chain topology, would result in intriguing, branched copolymers with the potential to be applied in nanomedicine. Therefore, we studied the self-assembly behavior of these copolymers in aqueous media, as well as their ability to form complexes with cationic proteins, namely lysozyme (LYZ) and polymyxin (PMX). Various physicochemical characterization techniques, including size exclusion chromatography (SEC) and proton nuclear magnetic resonance (^1^H-NMR), verified the molecular characteristics of these well-defined copolymers, whereas light scattering and fluorescence spectroscopy techniques revealed promising nanoparticle (NP) self- and co-assembly properties of the copolymers in aqueous media.

## 1. Introduction

Polymeric nanoparticles (PNPs) have manifested a widespread research interest in nanomedicine [1,2,3], including key pillars such as drug delivery [4], bio-imaging [5] and diagnostics [6]. The potential to overcome drug delivery barriers, as well as minimal drug bioavailability and stability or intensifying the potent localization ability of the drug to a specific biological target, has been established, utilizing an intriguing polymer class, amphiphilic copolymers [7,8,9]. Amphiphilic polymer chains, which comprise hydrophilic and hydrophobic segments connected by covalent bonds, present enticing self-assembly properties when studied in aqueous media. Self-assembly is a result of the interplay between different noncovalent interaction forces. Hydrogen bonds, steric effects and hydrophobic interactions are the generally accounted forces regarding amphiphilicity. Specifically, the hydrophobic nonpolar molecules of an amphiphilic copolymer tend to disrupt the hydrogen bonding network of the aqueous medium and self-aggregate. The resulting formations consist of micellar structures of hydrophobic cores surrounded by the hydrophilic segments that form a protective shell against polar water molecules [10]. These core–shell PNPs may comprise singular or multiple amphiphilic polymer chains and vary in size, morphology and properties, depending on a plethora of polymer chemical features and several environmental parameters. Hydrophobic–lipophilic balance (HLB), molecular weight, macromolecular topology, and architecture, polymer dissolution method and polymer concentration in the solution are some of these tunable parameters, which offer flexible adaptivity towards the development of PNPs [11,12]. Several reviews on amphiphilic PNP studies denote their promise as novel nanocarriers of both hydrophobic and hydrophilic bio-relevant substances [13,14,15]. Moreover, amphiphilic copolymers have been reported to present significantly lower critical micelle concentration (CMC) values when compared to other amphiphiles like surfactants, a phenomenon that suggests high stability upon limitless dilution [16].

Therapeutically active proteins and peptides, which have emerged as an attractive alternative for the treatment of numerous diseases [17,18], are a class of molecules that due to possible degradation, demand novel vectors such as PNPs [19,20]. Biocompatible methacrylate-based copolymers often consist of synthetic polyelectrolytes that contain charged units. They have the ability to form polyion complexes (PIC) with oppositely charged proteins [21,22]. Complexation occurs through an extra noncovalent force, electrostatic interactions, which are favored against aggregation enforced by hydrophobic interactions, since the latter are prompt to cause protein denaturation [23]. In vitro experiments on PICs from several polymer–protein pairs have exhibited extended protein half-life in blood [24] and enhanced protein loading and release properties [25]. Furthermore, amongst the aforementioned benefits of PNPs, the selection of the monomer units plays a crucial role and offers modifiability to the system.

The general breakthrough regarding reversible addition fragmentation chain transfer (RAFT) polymerization, and several other synthetic techniques under the umbrella of controlled living radical polymerization in recent decades, is responsible for the ability to prepare versatile polymers from a wide range of monomers [26,27]. In this work, hyperbranched poly (lauryl methacrylate-co-tert-butyl methacrylate-co-methacrylic acid), H-P(LMA-co-tBMA-co-MAA) copolymers were designed via a two-step facile process. Hyperbranched copolymers (HCs) of hydrophobic biocompatible monomer components, lauryl methacrylate (LMA) and tert-butyl methacrylate (tBMA), were synthesized by RAFT copolymerization with ethylene glycol dimethacrylate (EGDMA), a multivinyl monomer that introduces the desirable branching points [28]. Subsequently, the H-P(LMA-co-tBMA) precursor copolymers were partially modified via selective hydrolysis of the tertiary butyl segments to yield MAA segments. PMAA, bearing carboxylic groups, is a biocompatible weak anionic polyelectrolyte known for pH-responsive properties. The HLB of PMAA NPs can be tuned according to the pH value of the surrounding environment. In acidic conditions, due to protonation, MAA segments become more hydrophobic, whereas in basic conditions where ionization occurs, MAA units become more hydrophilic [29,30], a phenomenon normally associated with drug release [31,32]. The pK_a_ of MAA is in the range of five, therefore, in physiological media, it is in a hydrophilic state [33]. The study aims to investigate the self-assembly behavior of these novel materials, which appertain to a negligibly examined area of copolymers, where molecular amphiphilicity is randomly distributed upon a dense, highly branched macromolecular architecture and, furthermore, examine whether the combination of the unique architecture and chemical composition result in polymer structures which fulfill the requirements for the delivery of biorelevant proteins or peptides.

HCs have recently attracted research attention concerning PNP production and nanomedicine because of certain advantages when compared to their linear counterparts. To date, properties like improved thermodynamic stability [34], higher drug loading and release efficiency [35], as well as enhanced biocompatibility [36], have been reported. Additionally, HCs, and consequently the NPs emerging from their self-assembly, possess an increased surface area with multiple chain end groups readily available for functionalization [37]. Taking into consideration all the above, the hydrolyzed HCs (HHCs) were furthermore studied towards their ability to form electrostatic complexes with model cationic protein lysozyme (LYZ) and polymyxin (PMX) peptide in order to evaluate their potential as nanocarriers for bio-applications.

## 2. Materials and Methods

### 2.1. Materials

Monomers, tert-butyl methacrylate (tBMA), lauryl methacrylate (LMA), monomethyl ether hydroquinone or butylated hydroxytoluene (inhibitors) removers, 2,2 azobisisobutyronitrile (AIBN), 4-cyano-4-(phenyl-carbonothioylthio)-pentanoic acid (CPAD), pyrene, egg white lysozyme (LYZ), polymyxin B sulfate (PMX) and all solvents including 1,4-dioxane (99.8% pure), n-hexane, tetrahydrofuran (THF), trifluoroacetic acid (TFA), methanol, deuterated tetrahydrofuran (THF-d8) and deuterated chloroform (CdCL_3_) were supplied by Sigma Aldrich (St. Louis, MO, USA). Ethylene glycol dimethacrylate (EGDMA) monomer, utilized as the branching agent, was purchased from Merck (St. Louis, MO, USA).

### 2.2. Preparation of H-P(LMA-co-tBMA-co-MAA) Copolymers

HCs, with three varying compositions of tBMA and LMA, (H-P(tBMA-co-LMA)), were synthesized as precursor copolymers via a one-step RAFT polymerization. The detailed synthetic procedure is described below, giving information on the synthesis of HC 1 copolymer to exemplify the process. LMA (1.4 g, 5.5 mmol), tBMA (0.6 g, 4.2 mmol) and EGDMA (0.076 mL, 0.4 mmol) were first purified by inhibitor remover-packed columns. The inhibitor free monomers, radical initiator AIBN (16.4 mg, 0.1 mmol), chain transfer agent (CTA) CPAD (55.8 mg, 0.2 mmol), 8 mL of 1,4-dioxane (priorly dried over molecular sieves) and a stirring magnetic bar were placed in a 25 mL round bottom flask. The flask was sealed with a rubber septum and the reaction solution was degassed by nitrogen flow for about 15 min. The flask was then placed in an oil bath under magnetic stirring. Polymerization conditions were set as 70 °C temperature and 24 h duration. Polymerization was terminated by following the next steps. The flask was removed from the oil bath, and the mixture was completely frozen by placing it at −20 °C in a freezer for about 20 min. After freezing, the mixture was left to return to room temperature until finally being exposed to air. The received polymer solution was later purified from remaining monomers or oligomers by precipitation in hexane excess. The precipitate was dried in a vacuum oven for 48 h at room temperature and afterwards collected and stored in the fridge. The purified polymer was subjected to molecular characterization (Table 1) and, next, the hydrolysis reaction was carried out, in order to achieve the desired amphiphilic polyelectrolyte copolymer.

The hydrolysis of HHC 1 was accomplished as follows. We placed 1.3 g of HC 1 and 26 mL of THF in a 50 mL round bottom flask with a magnetic stirring bar. When the mixture became translucent, five times the equivalent of TFA (1.05 mL, 0.013 mmol), with respect to the molar mass of the tBMA units, was added to the mixture under stirring. The flask was sealed, covered with aluminum foil for light protection and left under stirring for 24 h at room temperature. The polymer was concentrated in a rotary evaporator and later was dried in a vacuum oven for 48 h, in order to isolate the solid material. Molecular characterization revealed that the conversion yield was dependent on the copolymer (Table 1); hence, HC 2 and HC 3 were furthermore hydrolyzed (for one week under the same conditions) to assess whether the hydrolysis was dependent on reaction time.

### 2.3. Preparation of Hydrolyzed Hyperbranched Copolymer Nanoparticles in Aqueous Media

An organic solvent displacement method [38] was utilized in order to prepare HHC NPs. In more detail, the HCs were first dissolved in THF. The organic solution was then injected into the aqueous medium under vigorous stirring, and, after one minute of stirring, the solutions were heated to eliminate the organic solvent. The aqueous medium selected was distilled water, where the ionic strength was fixed as 0.1 M. The final HHC concentration was 5 × 10^−4^ g/mL and the pH was seven.

### 2.4. Preparation of Hydrolyzed Hyperbranched Copolymers–Lysozyme Complexes in Aqueous Media

A series of five charge molar ratios of negatively charged HHC 2 or 3 and positively charged LYZ was produced by mixing different volumes of HHC and LYZ solutions in distilled water. The LYZ solution was prepared by direct dilution in water, whereas the 2.5 × 10^−4^ g/mL HHCs solution was prepared using the protocol described in Section 2.3, though without prior fixation of the ionic strength. Then, the HHCs solution was equally divided into five separate solutions and the proper amount of LYZ solution was injected under stirring; the five samples varied in LYZ concentration, while the HC 1 concentration was constant.

### 2.5. Preparation of Hydrolyzed Hyperbranched Copolymers–Polymyxin Complexes in Aqueous Media

Similarly to the HHC-LYZ complexes preparation, a series of three charge molar ratios of negatively charged HHC 1 or 3 and positively charged PMX were produced by mixing different volumes of HHC and PMX solutions in distilled water.

### 2.6. Toxicity Assay

An *Artemia salina* brine shrimp lethality bioassay was performed as an adaptation of the protocol proposed by Rashidzadeh et al. [39]. The upper limit of the salinity range (37 ppt) was used to prepare artificial seawater [40]. Dried cysts (700 mg) were suspended in artificial seawater, which was prepared with the dilution of NaCl in bottled mineral water (1 L). A 48 h incubation period at a temperature of 28 °C, under conditions of strong aeration was carried out. Fluorescent light was utilized for continuous illumination to conform the phototactic nature of the larvae (nauplii). Nauplii were added to the 96-well plates and incubated at 28 °C for 24 h. Each well held 250 μL of the medium, to which 10 μL of the polymer solution—corresponding to a 0.61 M final salt concentration—was added. The numbers of surviving nauplii in each well were counted under a stereoscope after 18 h and 24 h, based on the 10 s rule of no mobility. The control groups contained the same volume of artificial seawater. The experiment was conducted in fours for each concentration. The lethality was calculated using Abbott’s formula. The findings were then averaged to obtain the results reported.

### 2.7. Methods

#### 2.7.1. Size Exclusion Chromatography (SEC)

HC samples were passed through a Waters Corporation SEC set including a Waters 1515 isocratic pump, three µ-Styragel mixed pore separation columns of 10^2^ to 10^6^ Å pore diameter and a Waters 2414 refractive index detector equilibrated at 40 °C. The eluent consisted of a mixture of 5% *v*/*v* triethylamine in THF at a flow rate of 1.0 mL/min. The SEC column set was calibrated by linear monodisperse polystyrene standards of average molecular weights between 1200 and 152,000 g/mol, while Breeze 2 software was used for data acquisition and analysis.

#### 2.7.2. Proton Nuclear Magnetic Resonance Spectroscopy (^1^H-NMR)

^1^H NMR spectra of the copolymers were measured on a Varian 300 (300 MHz) spectrometer controlled by the VNMRJ 2.2C Software. Chemical shifts are given in parts per million (ppm) using tetramethylsilane as an internal reference, while spectra analysis was accomplished through MestReNova 6.0.2 Software.

#### 2.7.3. Attenuated Total Reflectance–Fourier Transform Infrared (ATR–FTIR) Spectroscopy

The FTIR spectra of the HC solid samples were recorded on a Bruker (Billerica, MA, USA) Equinox 55 Fourier transform spectrometer, equipped with a single bounce ATR diamond accessory (Dura-Samp1IR II by SensIR Technologies, Danbury, CT, USA). Spectra collected in the 5000 to 500 cm^−1^ range were the average of 64 scans.

#### 2.7.4. Dynamic Light Scattering (DLS)

DLS studies were conducted on an ALV/CGS-3 compact goniometer system (ALV GmbH, Hessen, Germany) with a JDS Uniphase 22 mW He–Ne laser, operating at 632.8 nm. An ALV/LSE-5003 light scattering electronics unit and an ALV-5000/EPP multi-τ correlator, including 288 channels, were used for stepper motor drive and limit switch control. Analysis of the correlation functions, which were recorded as the average of five measurements at a goniometer angle of 90°, was carried out using the cumulants method and the CONTIN algorithm. We utilized 0.45 µm hydrophilic PVDF and hydrophobic PTFE filters to remove any dust from aqueous and organic solutions, respectively. Samples were studied at C = 5 × 10^−4^ g/mL polymer concentration unless stated otherwise.

#### 2.7.5. Electrophoretic Light Scattering (ELS)

Zeta potential measurements were performed on a Nano Zeta Sizer Malvern Instrument (Malvern Instrument Ltd., Malvern, UK) with a 4 mW He–Ne, operating at 633 nm and a scattering angle of 173°. Results were recorded as an average of approximately 25 repeated scans and analyzed using the Smoluchowski equation.

#### 2.7.6. Fluorescence Spectroscopy

Fluorescence spectroscopy experiments were conducted on a Fluorolog-3 Jobin Yvon-Spex spectrofluorometer (model GL3-21) (HORIBA Scientific, Piscataway, NJ, USA). For the fluorescence pyrene assay used for critical aggregation concentration (CAC) determination, the emission spectra were collected in the 355–630 nm range, with the excitation wavelength being λex = 335 nm. HHC-pyrene mixed samples were prepared by the successive dilution of a stock solution, resulting in 11 copolymer solutions in the concentration range of 5 × 10^−4^ to 5 × 10^−9^ g/mL, in which the proper amount (1 µL/mL) of 1 mM pyrene solution in acetone was added. Each solution was then left in a dark place overnight for the evaporation of the acetone. Regarding experiments relative to HHC–LYZ complexes, where the tryptophan amino acid component is the fluorescence active residue, the emission spectra were collected in the range of 310–550 nm with λex = 290 nm as the excitation wavelength.

#### 2.7.7. Cryogenic Transmission Electron Microscopy (cryo-TEM)

Cryogenic transmission electron microscopy (cryo-TEM) images were obtained using a Tecnai F20 X TWIN microscope (FEI Company, Hillsboro, OR, USA) equipped with a field emission gun, operating at an acceleration voltage of 200 kV. Images were recorded on the Gatan Rio 16 CMOS 4 k camera (Gatan Inc., Pleasanton, CA, USA) and processed with Gatan Microscopy Suite (GMS) software version 3.31.2360.0 (Gatan Inc., Pleasanton, CA, USA). Specimen preparation was carried out using vitrification of the aqueous solutions on grids with holey carbon film (Quantifoil R 2/2; Quantifoil Micro Tools GmbH, Großlöbichau, Germany). Prior to use, the grids were activated for 15 s in oxygen plasma using a Femto plasma cleaner (Diener Electronic, Ebhausen, Germany). Cryo-samples were prepared by applying a droplet (3 μL) of the suspension to the grid, blotting it with filter paper and immediately freezing it in liquid ethane, using a fully automated blotting device Vitrobot Mark IV (Thermo Fisher Scientific, Waltham, MA, USA). After preparation, the vitrified specimens were kept under liquid nitrogen until they were inserted into a cryo-TEM holder Gatan 626 (Gatan Inc., Pleasanton, CA, USA) and analyzed in the TEM at −178 °C.

#### 2.7.8. Stereo Microscopy

A Leica S APO Stereo Microscope with a table stand was used for the observation of the *Artemia salina* brine shrimp lethality bioassay.

## 3. Results and Discussion

### 3.1. Hyperbranched Copolymers Synthesis and Characterization

**Scheme 1:**
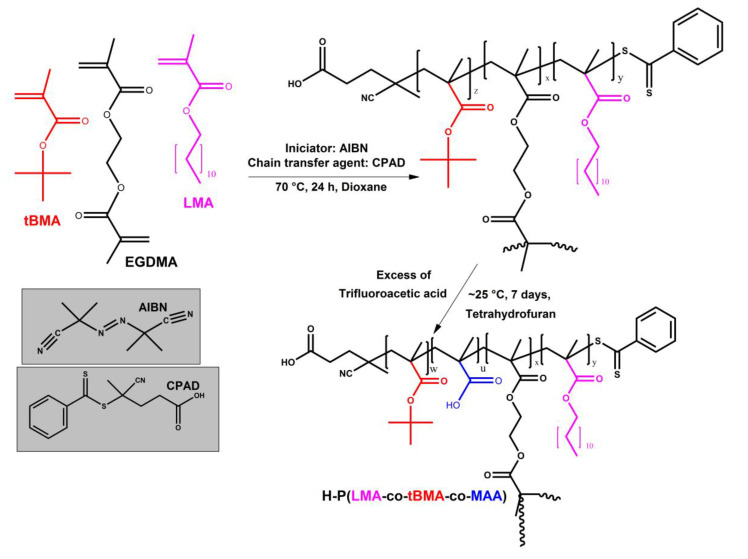
Synthesis of the HHC via RAFT polymerization and post-modification hydrolysis.

The targeted hyperbranched precursor copolymers (HC 1, HC 2 and HC 3) were obtained via the RAFT technique utilizing EGDMA as the branching agent (Scheme 1). The fabrication of hyperbranched polymers using controlled radical copolymerization of monofunctional monomers (one vinyl bond per molecule) and a judicious amount of bifunctional (two vinyl bonds per molecule) monomers is a reliable method to avoid unlimited chain propagation, which causes unwanted gelation. The CTAs in RAFT polymerization systems are responsible for suppressing radical concentration by creating stable radical intermediates. Intermolecular branching (crosslinking) and intramolecular branching (cyclization) are antagonizing factors in these chain growth systems, while different monomer units may be consumed at a separate pace, since LMA, EGDMA and tBMA molecules differ in flexibility and length. Additionally, it is known that once an EGDMA molecule joins a propagating chain via one of the two equally reactive vinyl groups, this results in reduced reactivity of the other pendant vinyl group due to steric hindrance [41,42].

**Figure 1:**
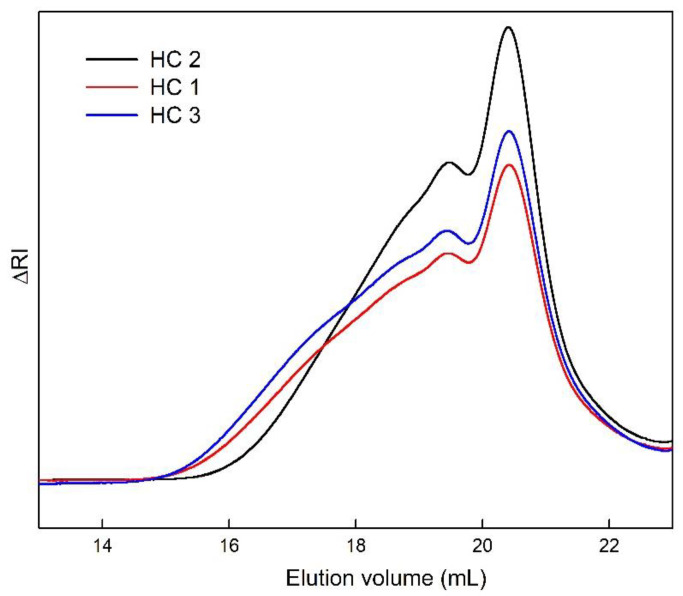
SEC traces of the precursor HCs.

**Figure 2:**
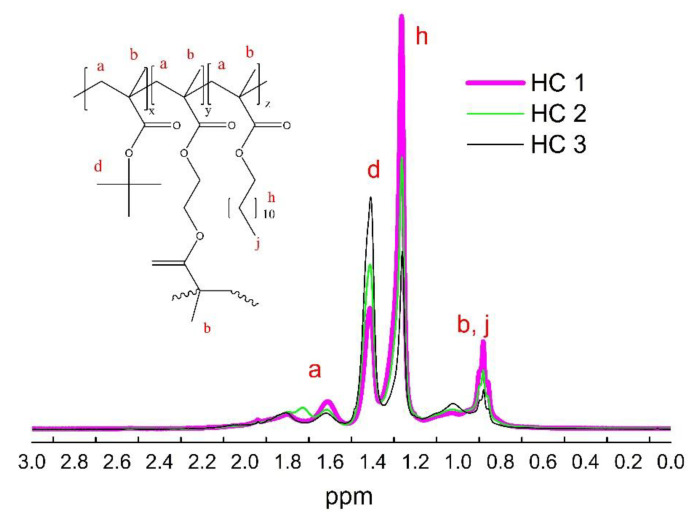
^1^H-NMR spectra of the precursor HCs (Hydrogen peak assignments are represented by the letters).

The peaks of the HCs’ SEC chromatographs portrayed in Figure 1 and the high solid product yield observed after precipitation (>85% in all cases) reveal the successful synthesis of three polymer species derived from an almost complete monomer-to-polymer conversion. The curves exhibit shoulders at a lower elution volume, which represent high molecular weight polymer chains. This phenomenon is likely attributed to cyclization side reactions and even some polymer chain knots formed during the process of cyclization. It is less likely that the dimethacrylates are consumed early and form hyperbranched microgels, since branching, meaning the bond formation with a pendant vinyl group on an already propagating chain, is generally known to occur at the late stage of the polymerization, while, additionally, such microgel structures would not be detected by SEC analysis [43]. The formation of these high molecular weight polymer chains does not seem to severely affect the homogeneity of the polymer products, since the polydispersity indexes (M_w_/M_n_), measured by SEC, are within the value range usually attained when the synthesis of hyperbranched copolymers is carried out using the RAFT technique [44,45]. Higher values of apparent weight average molecular weights (M_w_), compared to the theoretically calculated ones, are observed. Considering the fact that the SEC analysis system was calibrated by linear PS standards, the absolute M_w_ values are even higher than the ones measured, indicating that hyperbranched copolymers are formed. Regarding monomer distribution on the polymer chains, estimations based on ^1^H-NMR spectra (Figure 2) integrations revealed that the composition of the copolymers is almost identical to the monomer feed ratio.

**Figure 3:**
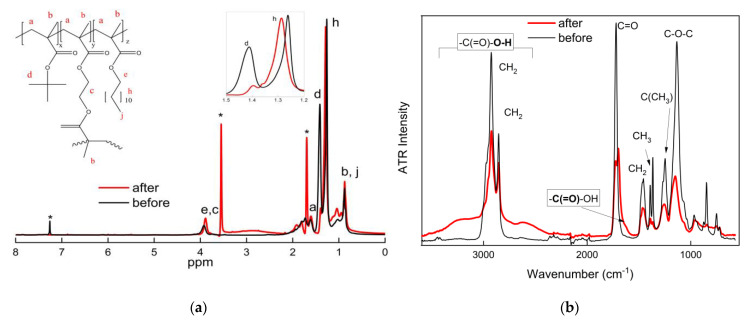
^1^H-NMR spectra in CDCl_3_ and d_8_-THF (**a**) and FT-IR ATR spectra (**b**) of HC 2 before and after 24 h hydrolysis (The letters in ^1^H-NMR spectra represent hydrogen peak assignments and the star (*) symbols are attributed to CDCL_3_ and d_8_-THF).

The copolymers were subjected to hydrolysis, targeting the total conversion of tBMA segments to MAA segments. The production of MAA copolymers is usually accomplished by synthesizing tBMA precursor polymers, since, normally, the RAFT copolymerization of unsaturated carboxylic acids is a challenging procedure [46]. Nonetheless, we have previously reported the copolymerization of MAA, LMA and EGDMA monomers via the RAFT technique, which resulted in polymers with analogous fidelity [47]. Nevertheless, we considered it interesting to examine the two-step process employing the hydrolysis of precursor copolymers in terms of synthetical aspects, including differentiations in the monomer distribution of the macromolecular polymer chains. Taking into account the hyperbranched architecture and relative steric hindrances within the polymer backbone, we assume that, in the latter case (two-step process), the three-dimensional globular chains may present a higher separation in hydrophilic and hydrophobic regions, since the butyl groups close to the chain ends in the surface of the hyperbranched “polymer sphere” are more accessible to hydrolysis. The hydrolysis reaction (Scheme 1) presented a significantly higher yield for the copolymers with a greater content of tBMA. Based on the comparison of the integrated intensity of the characteristic peak attributed to the tertiary groups in ^1^H-NMR spectra before and after hydrolysis, the reaction for a duration of 24 h yielded an over 85% conversion for HC 1, whereas less than 24% of HC 2 and HC 3 butyl groups were transformed to acid groups. Figure 3a shows the ^1^H-NMR spectra of HC 2 before and after hydrolysis. HC 1 and HC 3 hydrolysis were repeated for an extended period of 7 days which resulted in a 93% final conversion for HC 3, though no significant alterations for HC 1 (18% total) were observed. Given these results and the fact that relative linear polymers usually present almost complete conversion [48,49], the macromolecular hyperbranched architecture and especially the random distribution of monomers seem to drastically affect the yield of hydrolysis. A qualitative comparative study to confirm the post-modification was also conducted by FT-IR spectroscopy. FT-IR spectra of HC 2 in the solid state are shown in Figure 3b, where the broad band between 2500 and 3500 cm^−1^, attributed to the stretching vibrations of bonded hydroxyl functions of carboxylic acids, is prominent. Additionally, a slight shift of the peak near 1700 cm^−1^, assigned to carbonyl stretching vibrations, to lower wavenumbers is also visible, indicating the absence of a fraction of the butyl ester groups [49,50].

### 3.2. Self-Assembly Studies

**Figure 4:**
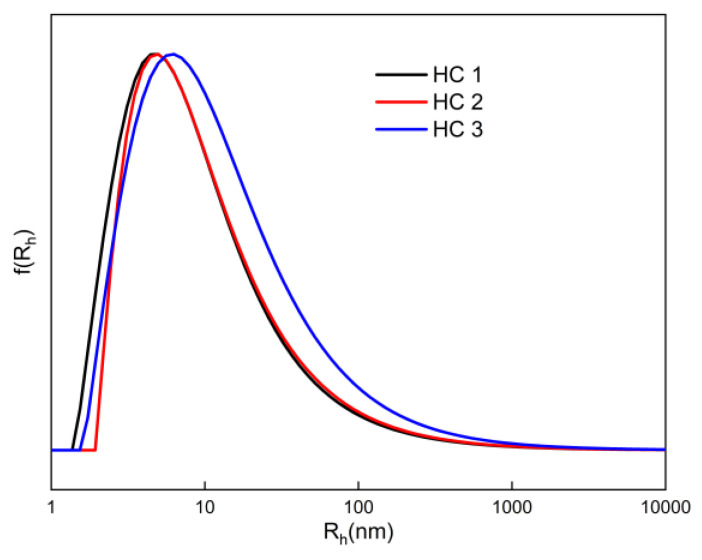
Size distributions from DLS analysis performed in THF at c = 10^−2^ g/mL for the different HC polymers.

The obtained HHCs are expected to present self-assembly properties in aqueous media due to their amphiphilic structure. Before studying HHCs solutions in aqueous environments, solutions of precursor HCs were investigated in a good organic solvent, THF, to determine the size of the dispersed unimolecular hyperbranched polymer chains. Even though the HHCs may display nonidentical dispersity to the precursor HCs because of their mass consistency, the precursor HCs were preferred for this investigation, due to the better affinity of tBMA and LMA than MAA units to the organic solvent. DLS measurements revealed monomodal and rather broad size distributions of macromolecules with similar average hydrodynamic radii (R_h_) for all HCs. The size distributions from CONTIN analysis are shown in Figure 4 and detailed results, including R_h_, scattered light intensity (I_90˚_) and size polydispersity indexes (PDI), are displayed in Table 2. The respective PDI values that almost exceed 0.5 in all cases are representative of the size dispersity of the globular hyperbranched polymer chains. These values are consistent with the M_w_/M_n_ dispersity values obtained by SEC.

**Figure 5:**
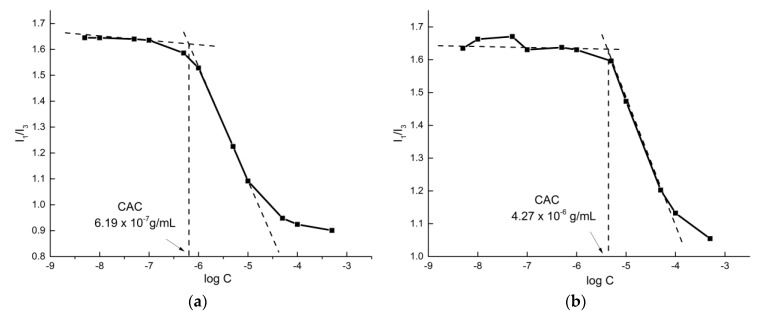
CAC determination for HHC 1 (**a**) and HHC 3 (**b**) copolymers.

The first step to examine the self-assembly properties of the HHCs in aqueous media is to determine the critical aggregation concentration (CAC) via the commonly employed pyrene assay, as described in the Section 2.7.6. The fluorescent properties of pyrene assist the tracing of hydrophobic domains in a polymer dispersion, since the emission spectra of the hydrophobic probe are characteristic of the polarity of the microenvironment they interact with. The fluorescence intensity ratio of the first (I_1_) to the third (I_3_) maxima of the emission spectra, as a function of HHC concentration for HHC 1 and HHC3, is shown in Figure 5. The intersection points between the two linear parts that define a sharp decrease in the intensity ratio correspond to the CAC value. CAC is affected by the hydrophilic–lipophilic balance (HLB) for chemically analogous copolymer species. As a general rule, copolymers with more hydrophobic content present lower CAC values [51]. This rule also seems to apply to the copolymers under study, since HHC 3, with 38% hydrophobic segments, presents an approximately six times larger CAC value than HHC 1, which consists of as much as 94% hydrophobic component. The presence of a CAC value intrinsically proves the ability of the HHCs to form micelle-like NPs. A value within the 10^−6^–10^−7^ g/mL range of polymeric micelles implies the thermodynamic stability of the assemblies [16]. The deviation between the I_1_/I_3_ values of the plateau after the transition area at higher concentrations indicates the formation of micellar NPs and also indicates that pyrene is located in a notably more hydrophobic environment, such is the case for HHC 1.

**Figure 6:**
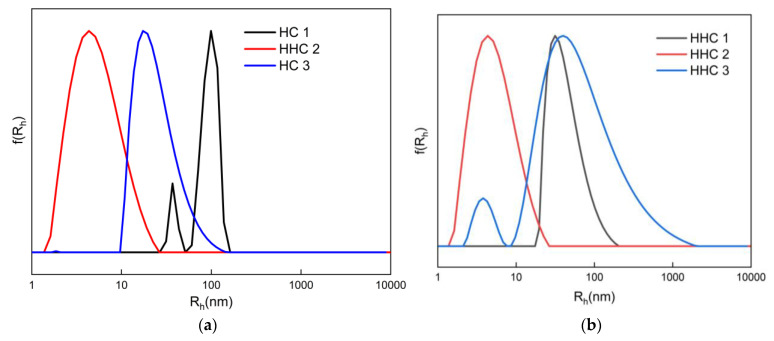
Comparison of size distributions from DLS analysis of the (**a**) HCs after 24 h hydrolysis in aqueous media and (**b**) the HHCs in aqueous media. (c = 5 × 10^−4^ g/mL).

DLS and ELS measurements were conducted in aqueous media to further analyze the HHCs’ self-assembly behavior and the structure of the formed aggregates. Figure 6b illustrates a comparison of the size distributions of the HHCs (c = 5 × 10^−4^ g/mL). The respective size polydispersity index and scattered light intensity values of the population are summarized in Table 2. The three HHCs, with varying HLBs, form NPs of low R_h_ values at the nanoscale (5–100 nm) with relative homogeneity. In more detail, only HHC 3 presents a distinct bimodal size distribution where a small (8%) population diverges from the main average R_h_. Given that, according to DLS results in THF, the single hyperbranched molecules R_h_ is around 10 nm, this small population characterized by an average R_h_ of 4 nm, could be attributed to some unimolecular micellar-type NPs. HHC 2 self-assembly also hints at the formation of unimolecular particles, since an average R_h_ (5 nm), similar to the HHC molecules in THF solution, is observed. The size decrease may represent the “shrinking” of the molecules due to hydrophobic interactions in the aqueous medium. Although amphiphilic hyperbranched copolymers are generally considered good candidates for the fabrication of unimolecular micelles [52], considering the unorthodox self-assembly behavior of random amphiphilic copolymers and mainly amphiphilic random copolymers with complex architecture [53,54], we examined whether much larger multimolecular aggregates that could not surpass the PVDF filters were formed. However, the reproducibility of measurements obtained from both filtered and non-filtered solutions of equal polymer concentration indicated no sign of this (Table S1 in Supplementary Materials). Additionally, the measured scattered light intensity presented low values attributed to NPs of low mass. In contrast, HHC 1 presents a population of NPs of multimolecular NPs (average R_h_ = 42 nm), which, according to the measured scattered light intensity, comprise much higher mass. This is expected in a system with such high hydrophobicity, as the HHC chains are densely packed in cores surrounded by the limited amount of hydrophilic chain domains which can form protective coronas. The formation of stable aggregates at such a high hydrophobic content should be attributed, at least partially, to the hyperbranched structure of the copolymers.

Samples of HC 1 and HC 3 after 24 h hydrolysis (4 and 16% hydrophilic MAA) were also studied by DLS for a comprehensive comparison (Figure 6a, Table 2). For both HCs, increasing the hydrophilic content using hydrolysis led to a significant decrease in the mass of the PNPs, meaning that the HHCs rearrange into a higher number of multimolecular NPs consisting of a lower number of macromolecules. This pattern of higher hydrophilic content leading to smaller size does not occur when comparing different HCs like HHC 2 and HHC 3, probably due to the influence of HC architecture. Moreover, HHC 2’s HLB may be the limit for the formation of unimolecular NPs, as higher hydrophilicity, as in the case of HHC 3, induces the formation of large but sparse aggregates. It is important to note that there was no sign of participation for the three systems with notably low hydrophilic content (4, 6, and 16% MAA), a fact that also supports the assumption that MAA units may be especially located on the outer surface of the polymers chains and, therefore, stable aggregates are favored.

The ζ_p_ measurements (Table 2) also lead us to two complementary observations. HHC 2 displays a very low negative surface charge that indicates the existence of NPs that are smaller in size, while HHC 1 negative surface charge is slightly higher than HHC 3, confirming the formation of higher mass aggregates.

**Figure 7:**
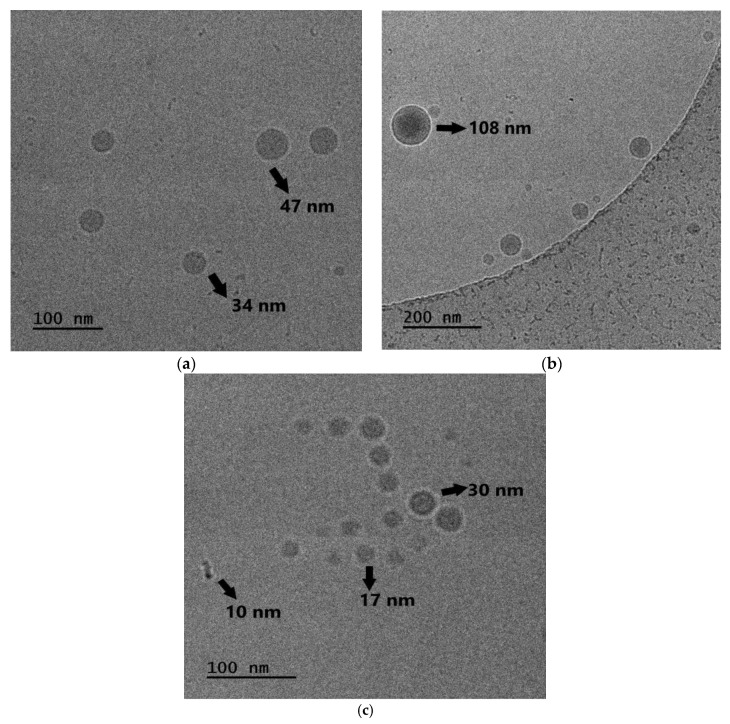
Cryo-TEM images from HHC 1 copolymer solutions showing NPs of (**a**) R_h_ ≤ 47 nm, (**b**) R_h_ ≤ 108 nm and (**c**) R_h_ ≤ 30 nm.

**Figure 8:**
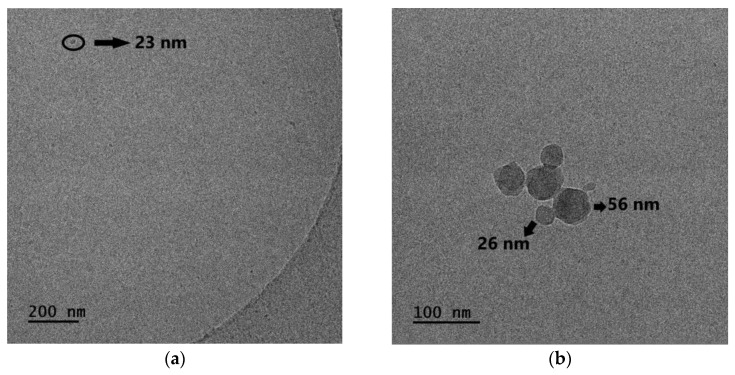
Cryo-TEM images from HHC 3 copolymer solutions showing NPs of (**a**) R_h_ = 23 nm and (**b**) R_h_ ≤ 56 nm.

Cryo-TEM was performed for further visual evaluation of the self-assembly properties of HHC 1 and HHC 3. Figure 7a–c and Figure 8a,b depict dried aqueous solutions of HHC 1 and HHC 3, respectively. The information available from the images is in accordance with DLS measurements. Quite spherical NPs are observed, with a calculated diameter D_h_ in the range of 10–108 nm for HHC 1 and 23–56 nm for HHC 3. In more detail, even though the majority of the PNPs abide by the range of the DLS histogram peak, few present significantly lower diameters. It is possible that, due to aggregation, which may look like overlaying in these kind of images (Figure 7c), some smaller NPs behave as larger aggregates during the DLS experiments. Similarly, this is possible for the NPs of HHC 3 in Figure 8b.

### 3.3. Protein/Peptide Complexation Studies

#### 3.3.1. Hydrolyzed Hyperbranched Copolymer–Lysozyme Complexation Studies

LYZ is a well-studied model cationic protein with known properties in drug delivery applications as a lytic enzyme. Its established antimicrobial properties, along with its available physicochemical characteristics, render it a valuable agent to achieve an insight into complexation mechanisms of anionic polyelectrolytes. Bearing an isoelectric point value clearly above physiological pH, LYZ carries eight positive charges per molecule; hence, many polymeric micelle complexes, stabilized via electrostatic interactions, have been reported [55,56,57]. We studied the formation of complexes in a series of negative (HHC) to positive (LYZ) charge mole ratios for HHC 2 and HHC 3 copolymers, while keeping the HHC concentration constant at 7.5 × 10^−5^ g/mL. Before discussing the results of the HHC–LYZ complexes, it is worth mentioning that the HHC solutions prepared in this case present different size distributions in the aqueous medium, where mainly multimolecular NPs—therefore, copolymer concentration and solution ionic strength—also play a significant role in the self-assembly behavior of the amphiphilic hyperbranched copolymers.

**Figure 9:**
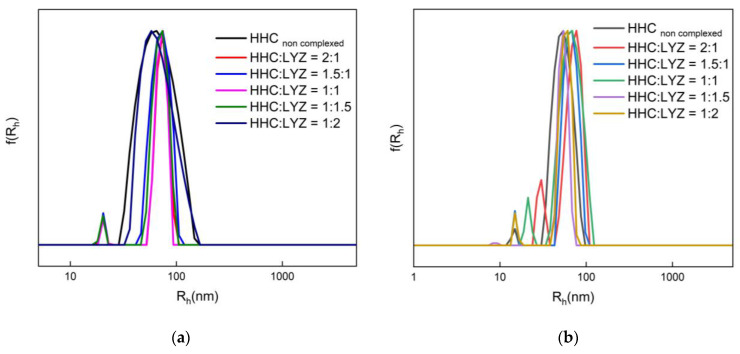
Comparison of size distributions from DLS analysis of the HHC–LYZ complexes obtained from (**a**) HHC 2 and (**b**) HHC 3 copolymers.

The DLS and ELS results, summarized in Table 3 (and Figure 9), signify that both HHCs can form complexes with LYZ via electrostatic interactions. A common observation for both systems is that the addition of the proper amount of cationic protein to the HHC solution leads to the formation of larger NPs in every case. However, an increase in LYZ concentration does not necessarily cause an increase in the measured scattered light intensity. Specifically, the LYZ–HHC 2 systems present equal or lower I_90_ values, whereas the LYZ–HHC 3 systems present a decrease in the value of I_90_ when the HHC:LYZ charge mole ratio is below or equal to 1:1 and an increase when the ratio is above 1:1. An explanation for the decreased intensity values is that the HCC NPs, once complexed to LYZ, are partially disintegrated, and form slightly larger and looser NPs, since LYZ is highly hydrophilic but also bears some hydrophobic domains [57], which may serve as an exceeding protective corona (while mainly electrostatic and some hydrophobic interactions take place between the components). This means that the polyion–LYZ complexes are formed after a rearrangement of the original HCC NPs. We tend to exclude the alternative interpretation that the vast majority of LYZ molecules are free in the solution and blanketed by the population of the HHC NPs. Though it is difficult to determine noncomplexed LYZ using DLS; a LYZ solution studied in various concentrations presented a size distribution of a main particle population around an average R_h_ of 2 nm (which is the reported measured size of the protein molecules [58]) and a small but very disperse population of particles above 100 nm (DLS data for neat LYZ samples are provided in Figure S1 and Table S2 of Supplementary Materials). None of these particles occur in any of the complexed systems; hence, few free LYZ molecules are expected to exist in the solutions of the complexes.

As an example, if we compare HHC 3:LYZ = 2:1 and HHC 3:LYZ = 1:2 complexes, assuming that the NPs do not rearrange, HHC 3:LYZ = 1:2, where LYZ concentration is four times higher than HHC 3:LYZ = 2:1, shows NPs of lower mass and size. This could only be attributed to the fact that the excess of LYZ molecules induces competition between hydrophobic and electrostatic interactions, causing the initially formed NPs to rearrange and expand, while a loose “cloud” of LYZ molecules surrounds the complex (an increase in hydrodynamic radius from R_h_ = 53 to 57 nm is observed). However, as mentioned above, the average mass is four times lower than the initial NPs; hence, it is more likely that the HHCP forms a higher number of smaller NPs after complexation with LYZ. Interestingly, a maximum increase in measured I_90_ and size, that usually happens when neutralization occurs in polyion complexes [59], is not observed for a charge ratio equal to one. Considering that the HHC and LYZ globular architectures will always lead to NPs, where some charged units may not be approachable, it would be difficult to detect a point where the neutralization of charges occurs. Additional information from ζ_p_ measurements (Table 3) suggests that complexation has surpassed the neutralization point, but this may be a consequence of the surface placement of LYZ molecules in the complexes.

In general, for these self-assembled systems, it is expected that multiple LYZ molecules interact with the surface of a single HHC NP, leading to the increase in the average R_h_ in some cases, as in HHC 3:/LYZ = 2:1, which is almost ten times the size of LYZ molecules. This could be attributed to further aggregation between NPs where bridges of LYZ molecules occur.

**Figure 10:**
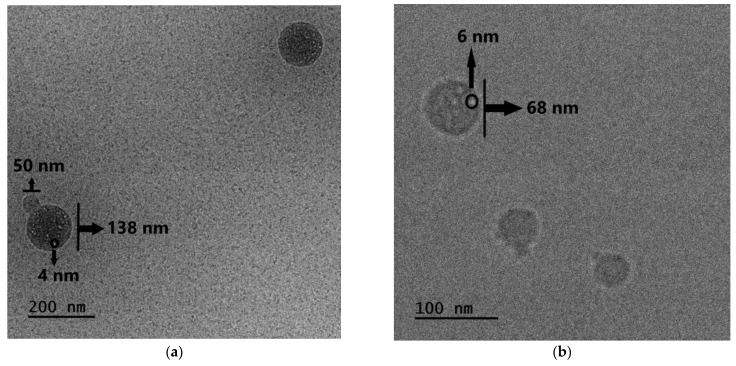
Cryo-TEM images from HHC 3:LYZ = 1:1.5 complexes showing NPs of (**a**) R_h_ ≤ 138 nm and (**b**) R_h_ ≤ 68 nm.

**Figure 11:**
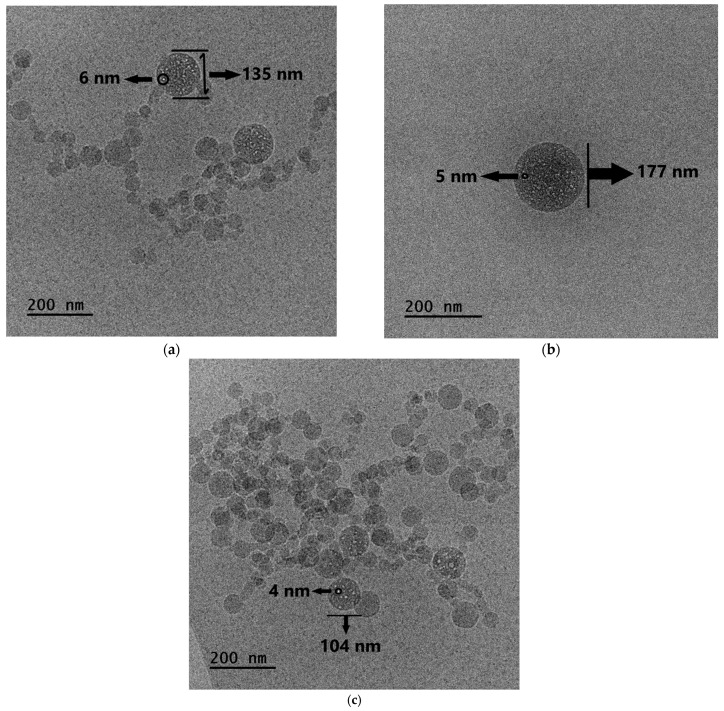
Cryo-TEM images from HHC 3:LYZ = 1:1 complexes showing NPs of (**a**) R_h_ ≤ 135 nm, (**b**) R_h_ = 177 nm and (**c**) R_h_ ≤ 104 nm.

This assumption is confirmed by cryo-TEM experiments, where depictions of the HHC 3:LYZ = 1:1.5 and HHC 3:LYZ = 1:1.5 complexes shown in Figure 10a and Figure 11a,b below indicate that multiple HHC NPs are interconnected. In particular, Figure 10a probably presents an example of two differently sized NPs, coupled as one, contributing to the increase in the mean R_h_, whereas the conformation of the NPs in Figure 11a may be attributed to LYZ bridges between the NPs. Based on the calculations from the images, the small white spots located on the surface of the NPs (that are not apparent in the cryo-TEM images of noncomplexed HHCs) could represent LYZ molecules. However, the possibility of small damage of the surface structure of the NPs due to high energy electrons could also explain these spots. All calculated D_h_ values are compatible with the size distribution range of DLS experiments.

**Figure 12:**
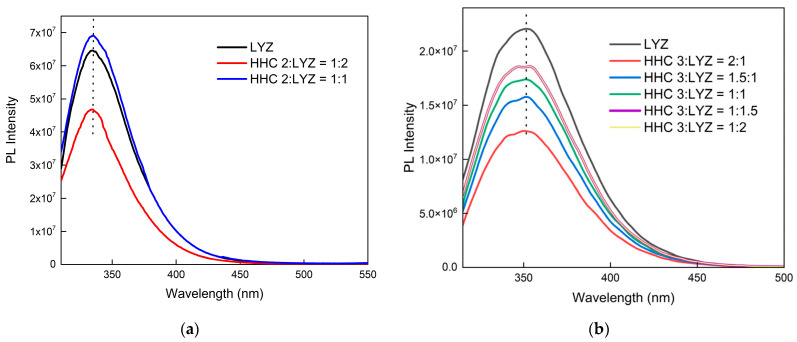
Fluorescence spectra of HHC–LYZ complexes for (**a**) HHC 2 and (**b**) HHC 3 copolymers.

In order to examine whether possible structural conformational changes in LYZ have occurred due to complexation, fluorescence spectroscopy was utilized for a comparison of the fluorescent properties between free and complexed LYZ molecules. Tryptophan amino acid residues, especially the two residues located at the substrate binding sites which are known to dominate the fluorescence emission of LYZ, constitute an indicative probe of LYZ integrity [60]. The emission spectra recorded for selected complexes are shown in Figure 12. The neat LYZ solutions studied are of equal LYZ concentration to the HHC:LYZ = 1:1 ratio complex for each HHC. Concerning maximum fluorescence intensity, it is observed that all mixed solutions, except HHC 2:LYZ = 1:1, present lower values than the neat LYZ solutions regardless of LYZ concentration. Surprisingly, while HHC 2:LYZ = 1:1 shows a slight increase, HHC 2:LYZ = 1:2 intensity is almost one and a half times lower. According to the literature, increased intensity may be attributed to the unfolding of the protein and exposure of tryptophan residues to a nonpolar environment, whereas decreased intensity is a sign of the quenching phenomena [61]. Also, a slight blue shift in the wavelength of the maximum emission intensity from 335 to 334 nm, which is also a marker of a nonpolar environment, is observed for HHC 2:LYZ = 1:1. A slight blue shift from 352 to 350 nm is observed for HHC 3: LYZ complexes too, although, contrarily, intensity seems analogous to the concentration of LYZ. The fact that evidence of a hydrophobic environment around tryptophan residues are more prominent in HHC 2 complexes, where the HHC NPs utilized for complexation consist of markedly higher hydrophobic content, implies that an interplay (interaction) between small hydrophobic domains in the interior of LYZ molecules and HHC hydrophobic domains is possible [61]. However, we doubt that any LYZ denaturation occurs, since the spectral variations between complexed and noncomplexed LYZ solutions are negligible. Overall, the results corroborate the conclusion that, besides electrostatic interactions, complexation may take place via hydrophobic interactions as well.

#### 3.3.2. Hydrolyzed Hyperbranched Copolymer–Polymyxin Complexation Studies

PMX is an antimicrobial peptide reintroduced in clinical research as a last-resort alternative for the treatment of infections caused by Gram-negative bacteria which are resistant to conventional antibiotics. It was formally discontinued from clinical use until the late 1980s, due to its neuro and nephrotoxicity. Currently, it is employed along novel engineering strategies to enhance its safe delivery, rather than exploring the time-consuming and as of now ineffective fabrication of novel multidrug formulations [62]. The amphiphilic and cationic nature of PMX is mainly responsible for its toxicity and therefore efforts to mask cationic groups while preserving the bioactivity are actively investigated. The usage of polyion complex NP delivery platforms is one of the strategies reported to optimize the antimicrobial activity of PMX [63,64].

**Figure 13:**
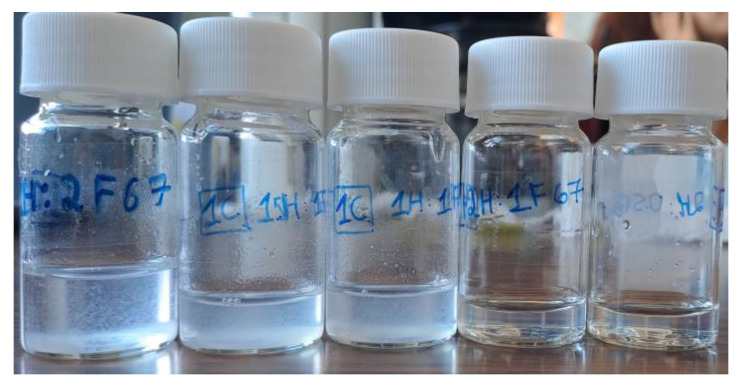
Photograph of solutions of HHC1:PMX complexes with a charge ratio of 1:2 to 8:1 (left to right).

Similarly to HHC-LYZ complexes, a series of different negative to positive charge molar ratios from 2:1 to 1:2 were initially prepared, though diluted samples of HHC 1 and HHC 3 solutions discussed in the self-assembly studies section were employed instead. In contrast to LYZ experiments, the HHC 3:PMX = 1:2, HHC 3:PMX = 1.5:1 and HHC 3:PMX = 1:1 ratios precipitated immediately after mixing, indicating strong electrostatic interaction between the components. Hence, the HHC 3:PMX = 4:1 and HHC 3:PMX = 8:1 ratios were chosen for DLS studies. Figure 13 is a photograph of the five solutions of HHC:PMX complexes in order of mention. PMX molecules are small cyclic chains of low mass (1.3 kDa) that carry five positive charges at physiological pH. PMX mobilization and dispersion of positive charges around negative charges may be more feasible than in the case of LYZ, where eight positive charges are distributed among the structure of a 14 kDa molecule. Equimolar amounts of negative and positive charged groups are therefore expected to form unstable suspensions that phase separate due to the higher possibility of the system towards neutralization. However, this is not applicable for HHC 1.

**Figure 14:**
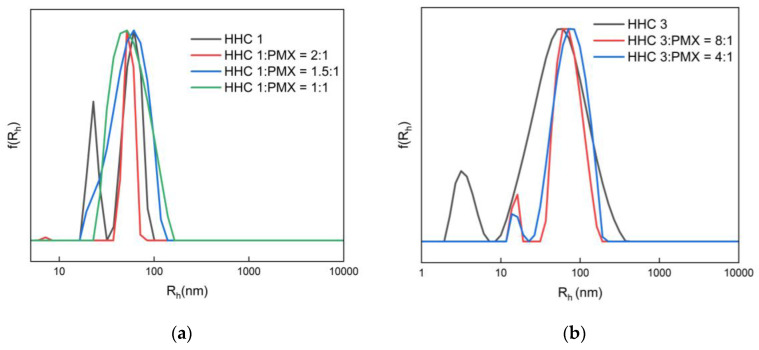
Comparison of size distributions from DLS analysis of the HHC–PMX complexes obtained from (**a**) HHC 1 and (**b**) HHC 3 copolymers.

Table 4 shows the results of DLS and ELS analysis of the HHC–PMX complexes, while size distributions from CONTIN analysis, in comparison to the neat HHC aggregates, are given in Figure 14. The results for the diluted neat HHC samples further prove that concentration and ionic strength strongly affect self-assembly. In the case of HHC 3, the two samples of equal concentration present NPs of similar size, although they are approximately 150 times lower in terms of mass, when the solution ionic strength is fixed initially. Generally, an increase in scattered light intensity is measured when PMX concentration increases and tends to counterbalance the negative charges. The decreased PDI values of the complexed NPs, in comparison to neat HHC NPs, suggest that the addition of PMX leads to more homogeneous particle populations, whereas it is not obvious whether the NPs rearrange or not. Considering ζ_p_ measurements, HHC 1, which has a significantly lower amount of MAA anionic segments does not seem to present any sign of precipitation at the theoretically calculated neutralization point. A possibility that has not been examined before is that the presence of carboxyl groups at the polymer chain ends, derived from the incorporated CTA segments, could also influence the charge balance. The absence of an increase in R_h_ in combination with the ζ_p_ values, which are closer to a neutral point, indicates that complexation via strong electrostatic interactions is taking place in these systems. On the other hand, the average R_h_ for HHC 3 complexes has a notable increase (from 52 to 75 nm), while the surface charge of the NPs is still strongly negative. This could be attributed to NP rearrangement and a wide excess of free negative charges, or to the formation of reverse vesicle aggregates, where some smaller-size HHC NPs may decorate the positively charged PMX molecules that surround a larger HHC NP.

### 3.4. Toxicity Studies

The *Artemia salina* lethality test is a well-established approach, which has been implemented over decades to obtain toxicity data for various medicinal formulations. These tiny crustaceans, also known as brine shrimp, are capable of swimming around freely in minuscule amounts of water. This ability is quickly diminished upon encountering toxic substances, causing shrimp to sink to the bottom, immobile [65]. *Artemia salina* have an outstanding ability to adjust to hypersaline conditions of 5–250 g/L salt concentration, a short life cycle, minimal storage requirements in the cyst stage, a high hatchability yield, a distinctively contrasting body color, a small body size which renders them convenient for small-scale aquariums and adaptability to a broad range of nutrient resources as non-selective filter feeders. This approach has been implemented on polymeric nanostructures and serves as a suitable substitute for the widely used MTT assay [39,66,67]. Lethality was calculated using the equation given below:

Lethality  =  [(Test − Control)/Control] × 100

The samples examined were HHC 1 and HHC 2, the more diverse HHCs in composition. It was found that, for HHC 1, the aggregates’ lethality was at 19.25%, whereas, for HHC 2, it was at 19.17%. Given that normally acceptable lethality levels for various NPs are around 10–30% [68,69], these values are relatively low, revealing that the HHC aggregates consisting of biocompatible monomers are non-toxic. The raw data utilized for the calculations are presented in Tables S3–S5 of Supplementary Materials.

## 4. Conclusions

The synthesis of hyperbranched amphiphilic polyelectrolyte H-P(tBMA-co-MAA-co-LMA) copolymers was successfully carried out by RAFT copolymerization of tBMA, LMA, and EGDMA monomers, followed by post-polymerization hydrolysis of tBMA side groups. The RAFT technique yielded high conversion polymers of rather narrow molecular weight distributions, while post-modification was time-dependent and strongly influenced by the chemical composition of the HCs. Self-assembly studies of the HHCs in aqueous media revealed that the HHCs can form unimolecular or multimolecular NPs, above a CAC value that suggests high thermodynamic stability. Furthermore, it was proven that, by altering parameters such as HLB, concentration or ionic strength, the self-assembly properties vary. The HHCs, bearing biocompatible monomers and polyelectrolyte units, were tested for the development of polyion complexes in the presence of the cationic bioactive/antimicrobial protein LYZ and PMX peptide. Driven by electrostatic and hydrophobic interactions, HHC complexes with LYZ and PMX, with small sizes and high homogeneity, were obtained. Lastly, the toxicity assay employed showed the biocompatibility of HHC aggregates in aqueous media. The above results allow us to propose these novel copolymers as potent delivery nanoplatforms for bioactive compounds. Moreover, along with our previous work [47], the ability of rather easily synthesized hyperbranched amphiphilic copolymers with random monomer distribution to self-assemble into functionalizable NPs is highlighted.

## Figures and Tables

**Table 1:** Precursor and final HCs’ molecular characteristics.

HC	Initial Feed Ratio ^c^	M_w_ ^a^ (g/mol) (×10^4^)	M_w_/M_n_ ^a^	wt% tBMA Theoretical	wt% tBMA ^b^	wt% tBMA ^b^ after Hydrolysis
1	21:27.5:2:1:0.5	2.9	1.78	30	33	27
2	35:1.95:2:1:0.5	3.4	1.93	50	48	7
3	49:12:2:1:0.5	3.5	1.94	70	67	5

^a^ Determined by SEC. ^b^ Determined by ^1^H-NMR in THF-d8. ^c^ tBΜA:LMA:EGDMA:CTA:AIBN mole ratio.

**Table 2:** Physicochemical characterization results of HHC copolymers.

HHC	DLS Results in THF			LS Results in Aqueous Media for the HHCs				LS Results in Aqueous Media after 24 h Hydrolysis			
	I_90˚_ (a.u.)	R_h_ (nm)	PDI	I_90˚_ (a.u.)	R_h_ (nm)	PDI	ζ_p_	I_90˚_ (a.u.)	R_h_ (nm)	PDI	ζ_p_
1	130	9	0.52	16,450	42	0.22	−52	55,000	97 (87%), 38(13%)	0.16	−54
2	136	10	0.48	30	5	0.47	−6	30	5	0.47	−6
3	160	11	0.52	186	63 (92%), 4(8%)	0.49	−49	1920	24	0.23	−45

**Table 3:** DLS and ELS results for HHC–LYZ complexes.

HHC	HHC/LYZ Ratio	C_polymer_ (g/mL)	C_lysozyme_ (g/mL)	I_90°_ (a.u.)	PDI	R_h_ (nm)	ζ_p_ (mV)
2	HHC _Non complexed_	7.5 × 10^−5^	-	7000	0.12	65	−22
2	HHC:LYZ = 2:1	7.5 × 10^−5^	3.26 × 10^−4^	6050	0.13	74 (95%)/ 20 (5%)	+26
2	HHC:LYZ = 1.5:1	7.5 × 10^−5^	4.35 × 10^−4^	7000	0.10	71	+26
2	HHC:LYZ = 1:1	7.5 × 10^−5^	6.52 × 10^−4^	6850	0.11	72 (95%)/ 21 (5%)	+28
2	HHC:LYZ = 1:1.5	7.5 × 10^−5^	9.78 × 10^−4^	6240	0.11	71 (96%)/ 20 (4%)	+25
2	HHC:LYZ = 1:2	7.5 × 10^−5^	13.04 × 10^−4^	5000	0.11	67	+25
3	HHC _Non complexed_	7.5 × 10^−5^	-	4330	0.13	53 (98%)/ 14 (2%)	−57
3	HHC:LYZ = 2:1	7.5 × 10^−5^	4.93 × 10^−4^	5900	0.14	72 (86%)/ 30 (14%)	+19
3	HHC:LYZ = 1.5:1	7.5 × 10^−5^	6.57 × 10^−4^	5050	0.14	66 (96%)/ 15 (4%)	+20
3	HHC:LYZ = 1:1	7.5 × 10^−5^	9.86 × 10^−4^	4880	0.16	67 (94%)/ 21 (6%)	+27
3	HHC:LYZ = 1:1.5	7.5 × 10^−5^	14.79 × 10^−4^	3560	0.15	54 (99%)/ 9 (1%)	+27
3	HHC:LYZ = 1:2	7.5 × 10^−5^	19.72 × 10^−4^	1876	0.16	57 (95%)/ 15 (5%)	+30

**Table 4:** DLS and ELS results for the HHC–PMX complexes.

HHC	HHC:PMX Ratio	C_polymer_ (g/mL)	C_polymyxin_ (g/mL)	I_90°_ (a.u.)	PDI	R_h_ (nm)	ζ_p_ (mV)
1	HHC _Non complexed_	7.5 × 10^−5^	-	1880	0.22	59 (73%)/ 23 (27%)	−47
1	HHC:PMX = 2:1	7.5 × 10^−5^	0.85 × 10^−4^	1470	0.13	54	+25
1	HHC:PMX = 1.5:1	7.5 × 10^−5^	1.13 × 10^−4^	1145	0.18	53	+23
1	HHC:PMX = 1:1	7.5 × 10^−5^	1,69 × 10^−4^	2112	0.15	56	+24
3	HHC _Non complexed_	7.5 × 10^−5^	-	29	0.59	56 (90%)/ 3 (10%)	−54
3	HHC:PMX = 8:1	7.5 × 10^−5^	2.46 × 10^−4^	790	0.25	72 (94%)/ 15 (6%)	−50
3	HHC:PMX = 4:1	7.5 × 10^−5^	4.99 × 10^−4^	1550	0.24	75 (96%)/ 15 (4%)	−47

## Data Availability

Data are available on request from the authors.

## Supplementary Materials

The following supporting information can be downloaded at https://www.mdpi.com/article/10.3390/ma16247702/s1: Table S1: DLS data for filtered and non-filtered samples of HHC 2; Figure S1: DLS results for neat LYZ samples of various concentrations; Table S2: DLS data for neat LYZ samples of various concentrations; Table S3 obtained data from lethality experiments for neat units; Table S4: obtained data from lethality experiments for HHC 1; Table S5: obtained data from lethality experiments for HHC 3.
