# Supplementary material for: Anionic Hyperbranched Amphiphilic Polyelectrolytes as Nanocarriers for Antimicrobial Proteins and Peptides
Source: Materials (Basel). 2023 Dec 18;16(24):7702. doi: 10.3390/ma16247702 (PMC10745097; doi:10.3390/ma16247702)
Supplement: Supplementary file 1 [file materials-16-07702-s001.zip › materials-2763884-supplementary.pdf]

Supplementary Information

Table S1: DLS data for filtered and non-filtered samples of HHC 2

HHC 2	I_{90° (a.u.)	R_h (nm)	PDI
Sample 1/FILTERED	30	5	0.47
Sample 2/FILTERED	42	8	0.49
Sample 1/NON-FILTERED	35	5	0.40
Sample 2/NON-FILTERED	32	5	0.48

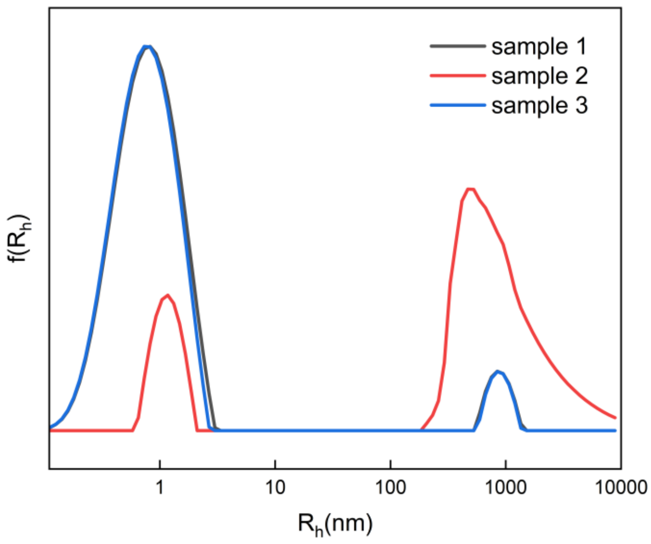

Figure S1: DLS results for neat LYZ samples of various concentrations.

Table S2: DLS data for neat LYZ samples of various concentrations.

LYZ	I_{90° (a.u.)	R_h (nm)	PDI
Sample 1/C=6.57×10^{-4} g/mL	19	1 (75%)/ 872 (25%)	0.47
Sample 2/C=13.04×10^{-4} g/mL	18	1 (24%)/ 831 (76%)	0.55
Sample 3/C=6.52×10^{-4} g/mL	15	1 (75%)/ 870 (25%)	0.41

The raw data utilized for the calculations regarding the toxicity assay are presented below.
A: number of tested units (shrimps)
B: number of survived units after 18 h
C: number of survived units after 24 h

Table S3: Obtained data from lethality experiments for neat units.

A	11	12	8	6	8	7	9	13
B	8	8	7	6	7	7	8	9
C	5	6	6	6	4	7	5	7

Table S4: Obtained data from lethality experiments for HHC 1.

A	11	9	7	5	7	7	9	14
B	9	6	6	4	6	5	6	9
C	5	4	6	2	6	5	5	9

Table S5: Obtained data from lethality experiments for HHC 3.

A	7	5	8	15	11	7	6	15	7	8	8
B	7	2	8	14	9	5	6	11	6	5	4
C	7	2	8	11	9	4	5	9	6	5	4
